# Application of Multi-Omics Approach in Sarcomas: A Tool for Studying Mechanism, Biomarkers, and Therapeutic Targets

**DOI:** 10.3389/fonc.2022.946022

**Published:** 2022-07-08

**Authors:** Zijian Zou, Wei Sun, Yu Xu, Wanlin Liu, Jingqin Zhong, Xinyi Lin, Yong Chen

**Affiliations:** ^1^ Department of Musculoskeletal Surgery, Fudan University Shanghai Cancer Center, Shanghai, China; ^2^ Department of Oncology, Shanghai Medical College, Fudan University, Shanghai, China

**Keywords:** sarcoma, genomics, transcriptomics, proteomics, metabolomics

## Abstract

Sarcomas are rare, heterogeneous mesenchymal neoplasms with various subtypes, each exhibiting unique genetic characteristics. Although studies have been conducted to improve the treatment for sarcomas, the specific development from normal somatic cells to sarcoma cells is still unclear and needs further research. The diagnosis of sarcomas depends heavily on the pathological examination, which is yet a difficult work and requires expert analysis. Advanced treatment like precise medicine optimizes the efficacy of treatment and the prognosis of sarcoma patients, yet, in sarcomas, more studies should be done to put such methods in clinical practice. The revolution of advanced technology has pushed the multi-omics approach to the front, and more could be learnt in sarcomas with such methods. Multi-omics combines the character of each omics techniques, analyzes the mechanism of tumor cells from different levels, which makes up for the shortage of single-omics, and gives us an integrated picture of bioactivities inside tumor cells. Multi-omics research of sarcomas has reached appreciable progress in recent years, leading to a better understanding of the mutation, proliferation, and metastasis of sarcomas. With the help of multi-omics approach, novel biomarkers were found, with promising effects in improving the process of diagnosis, prognosis anticipation, and treatment decision. By analyzing large amounts of biological features, subtype clustering could be done in a better precision, which may be useful in the clinical procedure. In this review, we summarized recent discoveries using multi-omics approach in sarcomas, discussed their merits and challenges, and concluded with future perspectives of the sarcoma research.

## Introduction

Sarcomas are rare malignancies with various subtypes, including more than 70 subtypes of soft tissue sarcomas (STS) and bone sarcomas (1). They are mesenchymal neoplasms with high level of heterogeneity, about 75% of which arise from soft tissue, 15% consist of gastrointestinal stromal tumors, and approximately 10% develop from bones (2). Although STS consists less than 1% of malignant tumor cases in the US (3), the sarcomas account for 15% of the childhood malignancies (4), manifesting the necessity of sarcoma research. The standard treatment for regional STS is surgery. Meanwhile, chemotherapy and radiotherapy serve as neoadjuvant or adjuvant treatment. When it comes to metastasis, chemotherapy may be the appropriate choice for palliative treatment (5). However, current treatments that use the same principle for all sarcoma subtypes have not taken the genetic differences into consideration. Novel therapies like immunotherapy and target therapy gradually come into play, focusing on the discrepancies that define each unique malignancy. Still, most of these precise therapies remain experimental attempts because of the shortage of studies, pointing out the significance of basic analysis for sarcomas.

There are plenty of omics studies of sarcomas, improving our understanding for the mechanism of the sarcomas from different angles. Each type of omics data provides information of the disease, assisting us in finding markers and exploring the biological pathways unique to the disease. For example, prognostic signatures using transcriptomic biomarkers have been established, including Complexity INdex in SARComas (CINSARC) (6), Genomic Grade Index (GGI) (7), and hypoxia-associated signatures (8). Three major situations—the limited effect of loci discovered, the influence from both coding region of genes and gene regulation, and the involvement of environment and genetic background—have made the power of single omics research unable to elucidate the whole picture of the disease development (9). This highlighted the importance of multi-omics approach in sarcoma.

The multi-omics strategies, combining genomics, epigenomics, transcriptomics, proteomics, and metabolomics, is an advanced research method in bioinformatics analysis, which provide us with detailed information for studies and better accuracy. Multi-omics provides a greater understanding of information of one disease, pointing out the direction for the discovery of the cause, the consequence, and the related interaction of the malignancy.

In this review, we provided an overview of multi-omics research for sarcomas in recent years, focusing on their approaches and results, thus discussing the significance of applying multi-omics approaches and providing insights into future perspectives of sarcoma research.

## Achievement with multi-omics approach

### Mechanism and Diagnosis

Precise diagnosis of sarcoma is not easy, considering its large number of subtypes and relatively small number of cases studied. Multi-omics provide a new aspect to dig deep into the mysterious field, and there have already been various multi-omics studies discovering details of tumorigenesis mechanism that is possibly capable of assisting the diagnosis. The results, the sarcoma subtypes, and the omics techniques applied in each study are summarized in **Table 1**. 

**Table 1:** Major results, sarcoma subtypes, and omics technologies of the multi-omics studies described in this review.

Tumor	Omics	Major Results	Ref.
OS	Transcriptomics and proteomics	*POLR3F* is a potential biomarker of OS.	(10)
OS	Transcriptomics and metabolomics	Four genes (*ENO1*, *TPI1*, *PKG1*, and *LDHC*) and two metabolites (lactate and pyruvate) form a signature of early detection.	(11)
OS	Genomics and transcriptomics	*TP53* rearrangements are the major mechanism of p53 inactivation in osteosarcoma that, with active microhomology-mediated break-induced replication and end-joining repair, contributes to the exceptional chromosomal instability in OS.	(12)
DSRCT	Genomics and transcriptomics	The most common imbalances were gain of chromosomes/chromosome arms 1/1q and 5/5p and loss of 6/6q and 16/16q. *GJB2* and *GAL* that showed higher expression in DSRCT compared with control tumors might be worthwhile to investigate as diagnostic markers at protein level.	(13)
LMS	Genomics and transcriptomics	Early mutations in *TP53* seem to exist in most LMS cases, whereas other mutations including *ATRX* deletions and Wnt/β-catenin alternations determine the genetic diversity of LMS.	(14)
LMS	Genomics and transcriptomics	LMS are characterized by inactivation of *TP53* and *RB1*, with recurrent alterations in telomere maintenance genes such as *ATRX*, *RBL2*, and *SP100*, and commonly display hallmarks of “BRCAness”.	(15)
UPSb	Genomics and transcriptomics	Recurrent somatic mutations in *TP53*, recurrent mutations in histone chromatin remodeling genes, including *H3F3A*, *ATRX*, and *DOT1L*, were identified in UPSb. *FGF23* can be a potential molecular biomarker for UPSb.	(16)
MIFS	Genomics and transcriptomics	The most common genomic rearrangements were breakpoints in or around the *OGA*, *NPM3*, and *FGF8* genes in chromosome band 10q24 and loss of 1p11-p21 and 10q26-qter. A breakpoint in or near *TGFBR3* in chromosome 1 was found. Amplification and overexpression of *VGLL3* was a consistent feature in MIFS and MIFS-like tumors.	(17)
DPFT	Genomics and transcriptomics	There is an age-associated differences in the origin of the *COL1A1*-*PDGFB* fusion and mostly arise after DNA synthesis. Transcriptionally upregulated genes in the amplified regions of chromosomes 17 and 22 were found, including *TBX2*, *PRKCA*, *MSI2*, *SOX9*, *SOX10*, and *PRAME*.	(18)
BCS	Genomics and transcriptomics	There is significant dysregulation of gene expression of epigenetic remodeling agents including key members of the PRC, Sin3A/3b, NuRD, and NcoR/SMRT complexes and the DNA methyltransferases DNMT1, DNMT3a, and DNMT3b.	(19)
OFS	Genomics and transcriptomics	RNASeq confirmed expressed mutations of *DICER1* and *NF1*. Amplification of *MYC* and deletion of *TP53* were found in CNV results.	(20)
UESL	Genomics and transcriptomics	Recurrent breakpoints and overexpression of the chromosome 19 microRNA cluster were observed, together with a *TP53* mutation or copy number loss.	(21)
HS	Genomics and transcriptomics	The PI3K pathway gene *PIK3R6* on chromosome 5 and upregulation of *TNFIAP6* in chromosome 19 were observed strongly associated to histiocytic sarcoma in dogs.	(22)
LMS	Genomics and transcriptomics	The promoter regions of *NPAS4* and *PITX1* genes were selected as the candidate methylation marker loci to distinguish uterine leiomyosarcoma and leiomyoma.	(23)
LPS	Genomics and transcriptomics	Dedifferentiated LPS had higher numbers of somatic copy number losses, amplifications involving Chr 12q, and fusion transcripts than well-differentiated LPS. *HMGA2* and *CPM* rearrangements occur more frequently in dedifferentiated components.	(24)
LPS, LMS	Genomics and transcriptomics	Upregulation of gene expression and gene copy number amplification of *MDM2* and *CDK4* were identified in LMS but not DDLPS. Upregulation of tumor related genes is favored in DDLPS, whereas loss of suppressor function is favored in LMS.	(25)
STS	Genomics and transcriptomics	More frequent *CDKN2A* and *CDKN2B* losses in post-radiation than in sporadic sarcomas. Recurrent *MYC* amplifications and *KDR* variants were detected in post-radiation angiosarcomas.	(26)

OS, osteosarcoma; DSRCT, desmoplastic small round cell tumor; LMS, leiomyosarcoma; UPSb, undifferentiated pleomorphic sarcoma of bone; MIFS, Myxoinflammatory fibroblastic sarcoma; DPFT, dermatofibrosarcoma protuberans family of tumors; BCS, BCOR-CCNB3 sarcoma; OFS, ovarian fibrosarcoma; UESL, undifferentiated embryonal sarcoma of the liver; HS, histiocytic sarcoma; LPS, liposarcoma; STS, soft tissue sarcoma.

Osteosarcoma (OS) lacks a sensitive and specific diagnostic marker, and most patients started their treatment in late stages. Integrating transcriptomic and proteomic data of OS cells, 13 biomarkers were chosen, in which *POLR3F* has most potential to be the diagnostic biomarker for early stages of OS (10). Another OS research integrated transcriptome and metabolome profiles and compared the difference between patients with OS and healthy individuals. Specific genes and metabolites were chosen on the basis of a set of criteria. After the pathway enrichment analysis, the glycolysis/gluconeogenesis pathway was found to be served as the only common pathway enriched in both omics. Finally, four genes (*ENO1*, *TPI1*, *PKG1*, and *LDHC*) and two metabolites (lactate and pyruvate) were chosen to form a signature as the predictive method for OS (11). Rearrangement of *TP53*, *RB1*, and *CDKN2A* was revealed in another study of OS, and a fusion of *PMP22* and *ELOVL5* was newly discovered. The involvement of non-homologous end-joining and microhomology-mediated end-joining DNA repair was confirmed by genomics. The lack of genomic evidence for 17 fusion transcripts, which were detected in the research, suggests a complex mechanism in trans-slicing. Most importantly, it was conformed that *TP53* rearrangement is the leading condition of inactivation of p53 in OS, the frequency of which is much higher in this study than previous statistics (12).

Desmoplastic small round cell tumor (DSRCT) is an aggressive malignancy that occurs mostly in male aged 15 to 35 years, and a *EWSR1-WT1* fusion gene could be found in most cases (27). The upregulation of *WT1* was confirmed in a study using RNA-seq, as well as other neural differentiation–related gene such as *GJB2*, *GAL*, *GALP*, and *ASCL1*. Meanwhile, the SNP arrays showed extensive copy number changes, the most frequent being gain of chromosome arm 1/1q and 5/5p and loss of 6/6q or 16/16q (13). The mechanism underneath needs further exploration.

Early mutations in *TP53* seem to exist in most leiomyosarcomas (LMS) cases, whereas other mutations including *ATRX* deletions and Wnt/β-catenin alternations determine the genetic diversity of LMS (14). In another study, inactivation of *TP53* and *RB1* appears to be a universal phenomenon, together with vast number of copy number variations (CNVs), mutational heterogeneity, and whole-genome duplication. Alternation of telomere maintenance genes like *ATRX*, *RBL2*, and *SP100* is observed as well (15).

A multi-omics study of undifferentiated pleomorphic sarcoma (UPS) of bone revealed recurrent mutations in *TP53* and histone chromatin remodeling genes. Eight somatic fusions were identified, including *CLTC-VMP1* and *FARP1-STK24* that were previously observed in multiple tumors. High expression of *FGF23* was revealed, which may serve as a biomarker of this subtype of sarcoma (16).

Myxoinflammatory fibroblastic sarcoma usually has the feature of translocation t(1;10) (p22-31;q24-25). However, this does not result in an expressed fusion gene; instead, the concomitant hemizygous loss of genes from proximal 1p and transcriptional deregulation of the FGF8 gene in 10q24 may be the reason for the biological process within the cells. In addition, regional loss of distal 10q and 3p and amplification of *VGLL3* showed a high frequency, which may promote the development of this disease (17).

Dermatofibrosarcoma protuberans is a rare sarcoma with a high local recurrent risk and a low metastasis rate. Research on the dermatofibrosarcoma protuberans family of tumors regarded the *COL1A1-PDGFB* fusion with a der(22)t(17;22) or ring chromosome as an age-related feature, and RNASeq identified regions with higher expression, validating former discovery of amplified genes on chromosome 17 and 22. These regions may also serve as therapeutic target point in the future (18).

BCOR-CCNB3 sarcomas, characterized with a BCOR and CCNB3 fusion gene, are round cell undifferentiated sarcomas that share morphologic and immunohistochemical features with Ewing sarcoma (ES). A multi-omics research was conducted for one male patient diagnosed with BCOR-CCNB3 sarcoma of the kidney. The mutation rate was relatively low, and no significant fusion was found besides BCOR-CCNB3. Differentiated gene expression analysis revealed multiple genes encoding key players of the *PRC2*, *PRC4*, *NuRD*, *NCoR*, and *mSIN3A* epi-genetic remodeling complexes, but few DNA methylation changes are correlated to changes in gene expression, suggesting the significant epigenomic dysregulation a consequence rather than a leading cause of tumorigenesis. Numerous changes in miRNA were observed, many of which have known relation with tumorigenesis (19).

Another case study focused on ovarian fibrosarcoma, a rare and aggressive type of sarcoma. The malignancy happened in a 9-year-old girl was diagnosed through morphology. The exome sequencing of both tumor and normal cells of the patient presented a low rate of mutations; when validated by RNASeq, expressed mutations discovered in previous studies were conformed, which are *DICER1* and *NF1*. Amplification of *MYC* and deletion of *TP53* were found in CNV results, which are commonly discovered in similar neoplasms (20).

Undifferentiated embryonal sarcoma of the liver exhibits recurrent breakpoints affecting the chromosome 19 microRNA cluster. Overexpression of this region was observed in these cases, together with a *TP53* mutation or copy number loss (21).

A multi-omics research on histiocytic sarcoma exhibits a special approach of tumor analysis. The study used flat-coated retrievers that frequently develop a disease of the similar histological and clinical features to histiocytic sarcoma. The PI3K pathway gene *PIK3R6* on canine chromosome 5 was observed strongly associated to histiocytic sarcoma in dogs, and upregulation of *TNFIAP6* in chromosome 19 is considered a risk factor as well (22). The research also shows the potential of dog breeds as excellent subjects for omics analysis, for they have perfect hereditary consistency after years of artificial selection.

Differential diagnosis is crucial for various malignancies, determining the subsequent treatment plans, and multi-omics approach surely casts a new aspect into this field. In an integrated study of genomics, epigenomics, and transcriptomics, Tomoko et al. compared the differences between leiomyoma and LMS in uterine, as a complement to histopathological diagnosis. Extensive chromosomal abnormalities were found in LMS, and eventually the promoter regions of *NPAS4* and *PITX1* genes were selected as the candidate methylation marker loci (23). Dedifferentiated liposarcoma (DDLPS) and well-differentiated liposarcoma are two types of sarcomas, the former frequently progresses to the latter. A study revealed higher copy number loss (CNL), amplification in 12q, and fusion transcripts in DDLPS. There were more rearrangements of *HMGA2* and *CPM* in DDLPS, which may influence the differentiation state of the LPS and need further study to explore (24). Another research focused on the difference between LMS and DDLPS. Through the bioinformatic analysis, amplification of regions encoding *MDM2*, *CDK4*, and *HMGA2* was significant in DDLPS, whereas mutations in *TP53*, *ATRX*, *PTEN*, and *RB1* appeared only in LMS. In addition, the microenvironment analysis showed more endothelial cells and fibroblasts in DDLPS than LMS (25). A comparison between post-radiation and sporadic sarcomas was conducted using genomic and transcriptomic strategies. Higher frequency of *CDKN2A* and *CDKN2B* was found in post-radiation sarcomas than the sporadic ones. Moreover, a high frequency of *MYC* amplification and 8% of *KDR* variants was found in accordance with previous research (26).

### Subtypes Clustering

Considering the vast amount of genomic data for malignancies, there are plenty of genetic differences among patients bearing the same type of sarcoma, sometimes making it possible for us to divide them into different subtypes with their respective clinical features and prognosis. Research showed that LMS subtype appeared in an early period of tumor and retained thereafter (14), enhancing the importance of subtype differentiation in malignancies of all stages.

Chondrosarcoma (CS) was divided into six molecular subtypes in a multi-omics research. Integrating DNA, mRNA, and microRNA data, three molecular features were found to influence the clinical outcome: a high mitotic state, regional 14q32 loss of expression, and IDH mutations, leading to genome-wide DNA methylation (28).

LMS are tumors with heterogeneous types, resulting in various prognosis. An analysis of 70 genomes and 130 transcriptomes of LMS observed three expression subtypes. Alternation of muscle related genes, including *DMD* and *MYOCD*, behaved differently in three subtypes, and deletions of the dystrophin gene may suggest an inferior outcome. Importantly, LMS molecular subtyping can be used to reveal its originating tissue, which may help adjust the treatment (14).

Rhabdomyosarcoma (RMS) was divided into two subtypes based on the existence of *PAX3/7* gene fusion. Mutation rates of somatic genes were relatively low in these tumors, especially for those with a *PAX3/7* fusion. As for the fusion negative ones, *RAS* mutation was activated in a large proportion, whereas, in a small part of them, the *BCOR* mutation was newly found (29). The subtypes were divided more precisely in another study. A1 and A2 subtypes, with alveolar histology and *PAX3/7* fusion, presented a worse prognosis. E1 and E2 has frequent CNVs and FGFR4/RAS/AKT pathway mutations and *PTEN* mutations/methylation, whereas E2 processes an extra p53 inactivation and a worse prognosis than E1 (30).

In a comprehensive analysis of sarcomas using RNASeq, CNV, and methylation data, four molecular subtypes (iC1, iC2, iC3, and iC4) with different clinical outcomes were identified, in which iC2 has the worst prognosis and iC4 has an enhanced immune state (31). Another research using The Cancer Genome Atlas (TCGA) database focused on six major types of sarcomas: DDLPS, LMS, UPS, myxofibrosarcoma (MFS), malignant peripheral nerve sheath tumor, and synovial sarcoma. The analysis defined three subtypes of DDLPS, uterine LMS, two subtypes of soft tissue LMS, and merged UPS and MFS as one subtype, whereas others have insufficient samples for further clustering. In addition, the pan-sarcoma analysis of omics data revealed frequent copy number alternations but few somatic mutations (*TP53*, *ATRX*, and *RB1*) in sarcoma, compared with other malignancies. The immune microenvironment analysis suggested its differential influence on different types of sarcomas (32).

### Prognostic Biomarkers

Prognosis is a vital variable in patients bearing the same type of sarcoma, yet we lack the powerful tool to anticipate the result. Multi-omics strategies unravel the inner differences between sarcoma cases, thus could partially reveal the possible outcome of them.

OS occur mostly in children and adolescents, which makes it important to identify the prognosis. Up to 25% of the OS cases resulted in metastasis, with pulmonary system being the major site (6). For patients with OS that had lung metastasis, molecules in MAPK signaling pathway and in the PI3K regulatory pathway were abnormally enriched (33). The urokinase plasminogen activator (uPA) is a serine proteinase known to involve in the metastasis of numerous solid tumors. The relationship between lung metastasis and uPA was studied in an integrated analysis using transcriptomic, proteomic, and secretomic analysis. The uPA/uPAR system regulated through autocrine and paracrine paths was found to be associated with metastatic behavior in OS and could activate other signaling pathways such as MAPK, which also promote the tumor metastasis. This makes activation of the uPA/uPAR axis a potential diagnostic marker for the conversion of OS cells from a non-metastatic to a metastatic phenotype, meanwhile indicating potential drug target for the prevention of metastasis (34). Another research of OS analyzed the genomic and transcriptomic information in cases with different overall survival. Integration of variation and survival data revealed six genes (*MYC*, *CHIC2*, *CCDC152*, *LYL1*, *GPR142*, and *MMP27*) to be the candidates for risk anticipation, and a risk score formulation was established to quantitively calculate the prognosis (35).

STS appears in various genetic forms, yet similarity could still be found in malignancies with unappreciable outcomes, which may serve as prognostic markers. Ribonucleotide reductase subunit M2 (RRM2) highly expresses in STS, and patients who possessed increased RRM2 level had worse overall survival. Overexpression of RRM2 induces proliferation, migration, invasion, and colony formation, whereas silencing of RRM2 arrests the cell cycle at G0/G1 phase and induces apoptosis (36). The result suggests that RRM2 could be a potential prognostic marker and a promising therapeutic target in STS. In another research, CNV of RNA regulatory genes were widely observed in STS patients, and there are possible relations between CNV and overall survival of patients. CNL of *METTL4* was associated with worse overall survival in both LMS and DDLPS. A low infiltration fraction of resting mast cells, which was found related to poor overall survival, was discovered in patient with LMS with CNL of *METTL4*. Facts above define CNL of *METTL4* as a potential prognostic marker for STS (37).

UPS is a subtype of STS with a commonly poor outcome, yet no biomarker is used at present to estimate the prognosis. Using two sets of transcriptomic data, high mRNA level of adenosine monophosphate deaminase 2 (AMPD2) was found correlated to a worse outcome. After comparing to CNV information, it was confirmed that CNVs promote AMPD2 expression in UPS. Together with the finding that AMPD2 may promote tumor growth and proliferation, AMPD2 could be a promising prognostic biomarker for UPS (38).

The expression level of replication-dependent histone mRNAs rises in DDLPS, compared with normal tissues, which is the result of the overexpression of *HMGA2*. The correlation between replication-dependent histone level and the aggressiveness of tumor makes it a promising prognosis marker (39).

E-cadherin protein expression was detected in LMS in a multi-omics study, which is also related to a better survival. The inverse correlation between E-cadherin repressor Slug expression and E-cadherin was also revealed, and in conclusion, the mesenchymal-to-epithelial reverting transition regulated by Slug appears to be a significant phenotype of LMS (40).

In a study of sarcomas, seven proteins related to overall survival (AMPKALPHA, CHK1, S6, ARID1A, RBM15, ACETYLATUBULINLYS40, and MSH6) were discovered using the proteomics data of different sarcoma subtypes. A prognostic risk signature was established and validated through transcriptomic profile (41). *ENO1*, *ACVRL1*, and *APBB1IP* were determined to be the prognostic biomarkers for sarcomas in another research, after comparing the four molecular subtypes divided on the basis of prognostic data (31). Another study used transcriptomic data to estimate the level of tumor-infiltrating lymphocytes. It was found that the infiltration of CD4^+^ T cell, influenced mainly by the CNVs, is related to the overall survival of sarcoma patients, especially for UPS (42).

### Treatment Evaluation

Currently, patients with sarcoma would receive surgical resection, radiotherapy, or systemic chemotherapy treatment as the standard routine. However, these may not be enough for patients with metastasis and recurrent situations. In fact, the 5-year survival rates of OS have not seen significant improvement in the past three decades (43). The ultimate purpose of medical research is to prevent and cure the existing disease, so the evaluation of drug efficacy is necessary. The omics approach focuses on the molecular level and may find the novel therapeutic biomarkers, especially for the targeted drugs.

CNV of *METTL4* in STS has a correlation with IC50 of 12 drugs including Temozolomide and Olaparib, suggesting a lower concentration need in clinical practice (37). Homologous recombination deficiency signature appeared in a large proportion of the LMS cases, as well as in other sarcomas, which may serve as a therapeutic biomarker, indicating the possibility of introducing PARP inhibitors (14, 15, 24, 44). An integrated study of DDLPS combined genomics, epigenomics, and transcriptomics data of four samples discovered a high frequency of *CEBPA* methylation. The methylation and silencing of miR-193b was found in patients bearing aggressive disease, suggesting its importance in liposarcoma genesis. Application of demethylating agents had an anti-proliferative and pro-apoptotic effect both *in vitro* and *in vivo* in the experiment, indicating a potential therapeutic method (45). Common receptor of tyrosine kinase/*RAS*/*PIK3CA* genetic axis was found in RMS tumors, which may be an option for targeted therapy (29). A study of DSRCT showed a low rate of somatic mutation but a large number of CNVs. Amplification of *FGFR4*, which is highly expressed in DSRCT cells, suggests a therapeutic potential and needs further study for a better comprehension of the mechanism within (46). Epithelioid sarcoma presented a high mutation rate and complex genomic characteristics. *SMARCB1* was the most frequent mutation, the loss of which leads to the aberrations in *SWI/SNF*, indicating its potential role in the abundance of translocations in epithelioid sarcoma. Because the *SWI/SNF* complex affects the proliferation of the tumor *in vitro*, there may be possibility for novel treatment (47).

Ginsenoside Rh2, a ginseng extract inhibiting the proliferation, migration, and invasion in multiple tumors, has the effect of anti-proliferation and apoptosis-induction in metastatic OS cells and inhibits migration by reversing epithelial-to-mesenchymal transition and promoting degradation of extracellular matrix (33). TRAF2- and NCK-interacting protein kinase (TNIK) was previously recognized as an essential factor of Wnt signaling pathway, whose inhibition leads to the loss of cell stemness. In a recent study, the suppression of OS cells was observed after applying TNIK inhibitor. Through transcriptomic analysis, pathways related to metabolism were upregulated, whereas pathways involved in Wnt signaling and pathways regulating the pluripotency of stem cells were downregulated, indicating the abrogation of OS stemness. Further metabolomics analysis discovered that drug-induced OS cells favored oxidative phosphorylation, unlike normal tumor cells that present aerobic glycolysis in a great extent (48). The result supports the potential of TNIK as a biomarker for molecular treatment, considering the fact that no current targeted therapy is approved for OS (49).

Immunotherapy shows promising effects in multiple tumors, but its attempts in sarcoma have not been desirable. Nine immune checkpoint-related lncRNAs (CD274, CD80, CD86, PDCD1 LG2, and LGALS9) were identified in a multi-omics research, the overexpression of which suggesting a worse outcome. Further investigation revealed their function of negative regulation in immune response, indicating a possible cause for the immunosuppressive environment in sarcomas, and may be a breakpoint in immunotherapy (50).

Metastasis is a common consequence of sarcomas, and a multi-omics study focused on the biological character of the metastatic sarcomas, trying to find a new therapeutic option. Structural variation and CNVs appeared to be the most frequent changes, and recurrent 17p11-12 amplifications were observed, suggesting a potential point for further research and treatment discovery (44).

To find drug efficacy biomarkers for childhood sarcomas, Brian et al. established tumor xenograft models representing most childhood malignancies. Models were tested using standard anti-tumor agents (vincristine and eribulin) and then studied with novel drugs (alisertib, volasertib, glembatumumab vedotin, and NTX-010). CNV and mRNA data of sensitive models were analyzed, and potential sensitivity biomarkers were discovered in most cases (51). In addition, these models are capable of further omics research in the future.

## Advantages and drawbacks of applying multi-omics approach

With the development of technology, precise and convenient research methods continue to alter the process of analysis, giving us insights into the depth of science. The omics approach collects mass quantity of data, analyzes the data with various mathematical methods, and finally recognizes abnormal biological features, the routine of which require much effort and time in early days, yet modern analysis software and advanced statistic formulas have made it easy even for analysis of multi-omics.

Multi-omics approach applies vast amount of data, compared with traditional sarcoma research. The integration of different types of omics data, combined with prior knowledge of regulation pathways, shows us a much more precise vision of activities inside sarcoma cells and may discover novel regulatory mechanisms as well. Unsupervised integrations identify every correlation among omics data, extracting biological process behind cancer progression, whereas supervised methods based on known phenotype knowledge improve the accuracy for cancer detection (52). These advanced research methods allow us to further study the complete picture of sarcoma as well as other malignancies, with accuracy, reliability, and comprehensiveness.

Another bonus point for multi-omics approach is the accessibility of data on the internet. The omics data take in almost the whole information of one aspect of a sample, which means great potential in mechanism discovery and feasibility for multiple analysis using different methods. On the basis of this idea, plenty of websites containing omics data had already been established. For example, TCGA, a database containing information of more than 30 human tumor types, catalogs aberrations in the DNA and chromatin of the cancer-genomes from thousands of tumors (53). There are other databases like Gene Expression Omnibus (GEO), Expression Atlas, and Oncomine, providing data in different aspects. Because of the low incidence rate of sarcomas, it is hard to obtain enough omics data for an integrated analysis, and such database provides an opportunity to gather data from all over the world so that a proper multi-omics research could be conducted. In fact, many of the studies above used information from online databases, as a supplement to their data of sarcoma cases. In addition, advanced technology has decreased the analytic burden with various computational algorithms like iCluster (54) and iOmicsPASS (55). Functional enrichment tools help cluster the variations through biological function, and online database equipped with these tools like Kyoto Encyclopedia of Genes and Genomes (KEGG) and Gene Ontology (GO) has made it easier for researchers to obtain the result. Because there are only few cases of sarcomas to be studied, the multi-omics approach provides a relatively efficient way to collect as much information as possible.

Although the advantages of multi-omics sarcoma research introduce convenience, there are deficiencies that need further improvement. The extremely low incidence rate of sarcoma brings obstacles for collecting abundant samples, not to mention their omics data. As a result, many of the multi-omics studies above used data from online databases, with little newly sequenced data from local patients, which may lead to bias because of the recurrent utilization of same sets of data. For those that only use samples from local patients, some of them were even not able to reach the standard of biomarker distinguishing because of the improper size of data. To obtain results with more stability and reliability, larger sets of omics data of sarcomas are needed. Another vital problem comes from the constitution of sarcomas. Although the therapeutic principle is “one-size-fits-all” in recent treatments, the inevitable fact that sarcomas variate in numerous subtypes should be taken into consideration when introducing precise medicine. As another result of insufficient data, several present studies roughly put all STS subtypes in one analysis, regardless of their genetic differences.

The expense of research should be discussed as well. Obviously, the omics study obtains much more information from patients’ tissue than traditional methods, despite the rise of cost. However, the omics approach did not come into stage with accuracy only. Technological advances, like expression quantitative trait loci, had made it possible for cost-efficient, high-throughput analysis of molecules (5).

The establishment of omics databases promotes the development of multi-omics study in a large extent, yet there are problems need to be fixed as well. Several databases containing information of different sources provide an opportunity for analysis of large number of samples, except for one question: the lack of a statistical standard. For example, in GEO database, there are data of progression free survival for each patient with UPS, whereas only time of death could be found in TCGA (41). Blindly combining data of distinct statistical standards could lead to conclusions with vital error; therefore, the universal standard for omics data collection is in urgent need. In addition to lack of a statistical standard, batch effects exist between different databases and sequencing data, which is also a problem to be solved in omics research.

## Future perspectives and conclusions

The use of multi-omics in sarcoma research has just started, and there are methods and process that could be improved, but the future application of multi-omics approach indeed has promising value. The systematic analysis of omics data can characterize the intersection of different level of information, decipher the molecular mechanism in tumor occurrence and development, and possibly anticipate the evolutionary process of specific cells. The sarcomas, many subtypes of which have complex karyotypes (32), are suitable for such analysis to explore the underlying characteristics. With the advanced analytical methods, molecular modeling algorithms, and computational tools, more could be discovered in diagnosis, prognosis, and treatment guidelines.

As we discussed above, the lack of sarcoma cases makes it difficult to conduct a proper analysis with enough data. Studies with larger sample sizes are required, which was mentioned by many of the multi-omics analysis of sarcoma. However, because of the low incidence rate of sarcomas, it is merely possible for one institute to gather enough sarcoma samples with sufficient omics data. The online database with a universal statistical standard may be one solution for this problem. The omics data, each representing the whole biological information of one level of cancer cells, contain abundant knowledge that could be repeatedly explored using different methods, which makes it valuable material that worth reserving for future research. Assembling information from several institutes may establish an aggregation capable of analysis for different types of sarcomas and, with the continuous update of analytic strategies and persistent expansion of data size, frequently reach novel discoveries.

**Figure 1:**
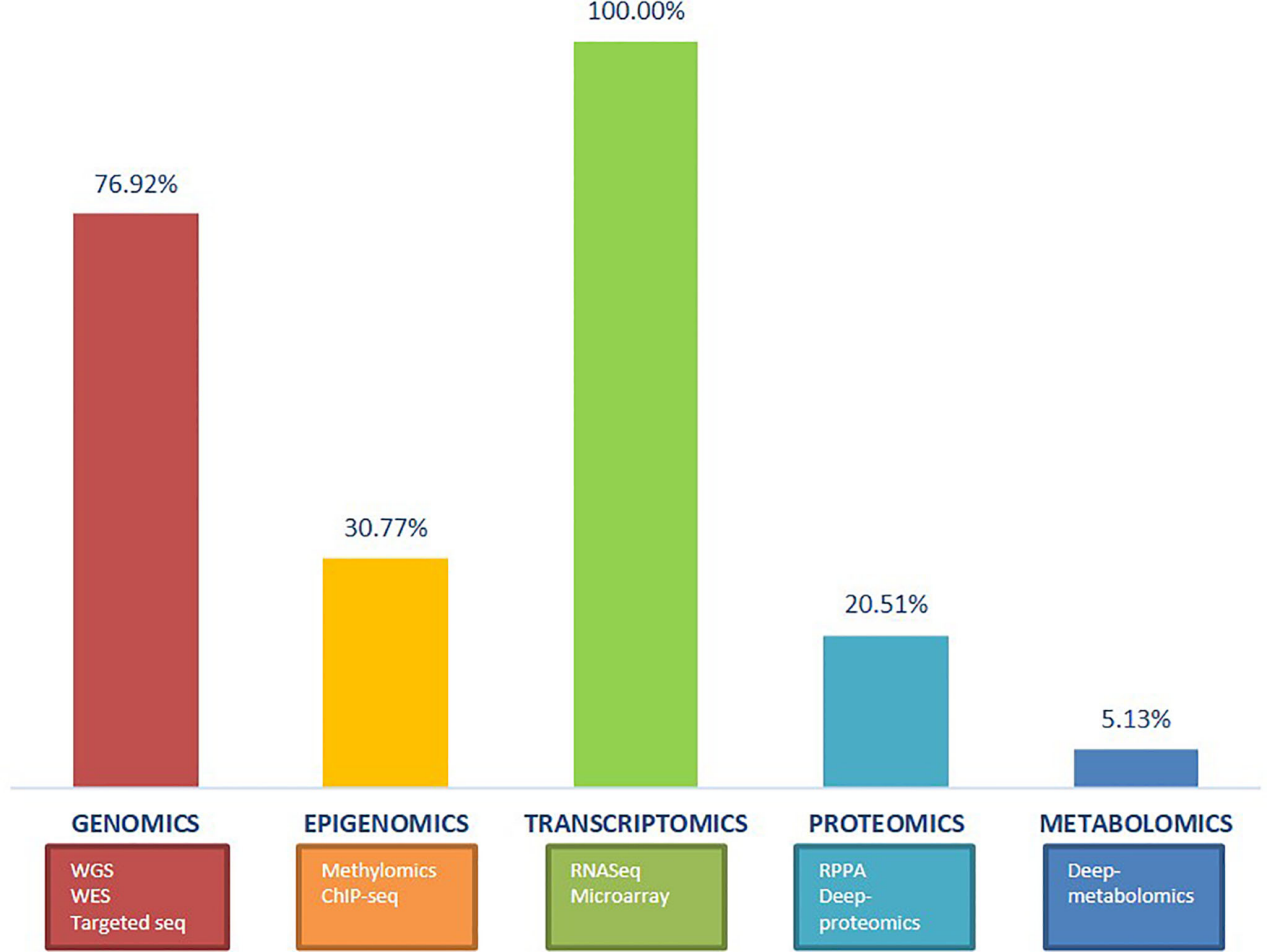
The frequency of use for each omics technologies in the multi-omics studies mentioned in this review. The frames below list the common methods of each omics.

Among the multi-omics studies on sarcoma, genomics, epigenomics, transcriptomics, and proteomics were widely applied on the basis of their respective demands, but few of the multi-omics research had considered metabolomics until recently (**Figure 1**). One reason for this phenomenon is the relative sparseness in knowledge of sarcoma metabolome, which act as an obstacle for the metabolomics study. Another reason may be the fact that the exploration of metabolomics of all tumors is still on the beginning point at present. Tumor cells survive in a reorganized, stressful, and metabolically competitive environment, forcing them to adapt to available metabolites for the reservation of cell plasticity (56). The metabolomics, evaluating metabolites in biospecimen for a better understanding of the full view of metabolism in malignancies, may lead to the discovery of new biomarkers and targets for advanced treatment. Sarcomas, displaying abnormal metabolic activity patterns, still need much efforts of exploration to link these situations to specific gene mutations (57). Common step of metabolomics analysis includes sample collection and preparation, metabolite extraction, data acquisition, analysis, and interpretation, and there are already results for OS in metabolic biomarkers of diagnosis, chemoresistance, and metastasis (58). When integrated with other omics data, there would be a better understanding of the metabolic pathways from the gene level.

In sarcoma research above, plenty characteristics of different omics were discovered (**Table 2**). Several types of sarcomas exhibit relatively low rate of somatic mutation, and such phenomenon was observed in some of the multi-omics studies, whereas a large number of CNVs were discovered, along with structural mutations and DNA methylation (13, 19, 20, 23, 29, 32, 37, 42, 46). The few tumorigenic driver genes, together with the genetic amplifications and deletions, construct an environment suitable for the development of malignancy. In addition, processes like trans-slicing altered the protein expression, contributing to the complexity of sarcoma cells. Such results reveal the shortage of traditional genomic research, which focuses mainly on somatic mutations and exaggerates the dominance of DNA sequence. Multi-omics studies on these sarcomas should probably put more attention on the CNVs and DNA methylations and capture their inner relations, which could lead to a more complete picture of sarcomas.

**Table 2:** Alternations of omics found in research of major sarcoma subtypes.

Tumor	OS	CS	DSRCT	LMS	RMS	DDLPS	UPS
MicroRNA Alternation		miR-27B, miR-125A, miR-140, miR-154, miR-382, miR-384		miR-181b-5p		miR-193b	miR-100-5p, miR-194-5p
Overexpression	ENO1, TPI1, PKG1, LDHC, SMARCA2, BAZ2A, POLR3F, CYC1, PITPNC1, PDCD2, DKK3, HS2ST1, UCHL3, DNASE2, WDR12, SKIP		FGFR4	ARL4C, CASQ2, LMOD1	PTPN11, ATM, ZNF350, TRPC4AP, FOXO1, ARID1A	MDM2, HMGA2, CDK4, GINS4, BRCA2, XRCC2, RAD51AP1, RAD51, RAD54B, XRCC1, POLQ, FEN1	FGF23
Gene Fusion	PMP22-ELOVL5		EWSR1-WT1	HMGA2-CPM	PAX3/7–FOXO1		APOL1-MYH9, PKNOX2-MMP20, ASAP2-ADAM17, CLTC-VMP1, FARP1-STK24
Structural Variation	TP53, RB1, CDKN2A, MDM2, MTAP			PTEN, BRCA1/2, ATM, FANCA, CHEK1, XRCC3, CHEK2, RAD51, FANCD2	CDKN2A, MIR17HG, CNR1, ERBB4, RPTOR, FRS2, CACNA1A, NRG1, FOXP2		
Methylation				PITX1		CEBPA	
Copy Number Variation	RB1, CDKN2A, CLU, BNIP3, IGFBP3, SPARC, TPD52, MEST, PRG1, OXTR, LOXL2, PTGFR, LYL1, DLG2	CDKN2A	GAL, GALP, ASCL1	TP53, RB1, CDKN2A, PTEN, ATRX, RBL2, BRCA1/2, ATM, FANCA, ALK, FGFR2, LIFR, PAX3, CDX2, SUFU, CDH1, DMD, MYOCD, DNMT3A, KAT6B, FLT3, FOXO1,	TP53, CDKN2A/B, MYCN, MDM2, MET, ALK, FGFR4, STAT6, IGF1R, MIR17HG, FRS2, MYOD1, CNR1	TP53, CDKN2A, MDM2, TERT, HMGA2, CDK4, ATRX, YEATS2, NF1, FRS2, NAV3	RB1, CDKN2A, MDM2, ING1, MYC, PDGFRA, KIT, KDR, PDGFA, PDGFB, VEGFA, CCNE1, YAP1, VGLL3
Mutation		IDH1/2, COL2A	TP53, TERT, ARID1A, HRAS	TP53, RB1, PTEN, PSDM11, CASP7, XPO1, SETD7, MTOR, ATRX, TOPORS, ATR, TP53BP1, TELO2, KMT2C, MAPK14, DUSP10, ZFP36L1, SRSF5, MED12, FH, MEF2C, HIST3H3, LAMA4	TP53, BCOR, CCND2, ARID1A, NRAS, KRAS, HRAS, FGFR4, PIK3CA, CTNNB1, IGF1/2, FBXW7	HERC2, MAPKAP1, HDAC1, DAZAP2, PTPN9	TP53, ATRX, H3F3A, DOT1L
Ref.	(10–12, 35)	(28)	(13, 46)	(14, 15, 23, 25, 32)	(29, 30)	(24, 25, 32, 39, 45)	(16, 32)

OS, osteosarcoma; CS, chondrosarcoma; DSRCT, desmoplastic small round cell tumor; LMS, leiomyosarcoma; RMS, rhabdomyosarcoma; DDLPS, dedifferentiated liposarcoma; UPS, undifferentiated pleomorphic sarcoma.

The research process of *TP53* gene fully embodies the value of the application of multi-omics. *TP53* is the gene of a transcription factor that induces gene involved in cell cycle arrest, apoptosis, and metabolism, thus playing an important role in the development of tumors. The majority of *TP53* mutations are missense mutations, which also gains oncogenic functions, leading to a worse outcome (59). The mutation rate of *TP53* was thought to be relatively low in previous studies (60), because only methods like exome sequencing were conducted on sarcoma samples. However, more alternations of *TP53* pathway in sarcomas were found in recent studies (12, 15, 16, 20, 21, 25, 29, 30), including genomic rearrangements, CNVs, expression regulations, and, in some cases, alternation of *TP53*, leading to a worse prognosis. This indicates the nonnegligible effect of *TP53* in sarcomas, and the proportion of *TP53* alternation of all kinds in sarcomas could be larger than previously expected. More should be studied in *TP53* in sarcoma research, and with the help of multi-omics, we could reveal the details of whole *TP53* regulation axis, figure out the influence of alternation in each step, and finally come up with methods to treat, to predict, and to prevent the deterioration of *TP53*-related sarcomas.

Many of the studies used multiple biomarkers to form a prognostic formula, which beats the single-biomarker prognosis considering the complexity of sarcoma tumorigenesis. However, some attempts of personalized treatment using genomic data did not come up with good results, indicating that genomic only is not enough for the precise prediction of therapy response (61). One of the studies uses a signature integrated with gene and metabolite levels (11), which could be a promising aspect of research, especially for multi-omics research. However, the application of such strategy needs thorough understanding of pathways involved in tumorigenesis, which is yet a relatively undeveloped field for sarcomas. Still, integrated signature could definitely fill the gap caused by differential expression and lead to a prognosis with lower false rate in the future.

Recent advances in high-throughput technologies have enabled the large-scale data analysis in genomics as well as other omics fields, which revolutionize the approaches in medical studies. Sarcoma research with the multi-omics strategies has reached various novel results, yet the vast unexplored field of unsolved issues needs further studies to fill in the blank. The integration of omics data, paving the way to a deeper understanding of the characteristics of sarcomas, may lead us to a more precise diagnosis, a more accurate prognosis, and more potential biomarkers for novel drug treatments.

## Author Contributions

ZZ and WS wrote and edited this manuscript and created tables and figure. ZZ, WS, YX, WL, JZ, XL, and YC reviewed and revised the manuscript. WS and YC provided direction and guidance throughout the preparation of the manuscript. All authors read and approved the final manuscript.

## Funding

This work was supported by Shanghai Science and Technology Development Funds (19411951700), the Lingang Laboratory (Grant No. LG-QS-202205-11), and the national natural science foundation of China (81802636).

## Conflict of Interest

The authors declare that the research was conducted in the absence of any commercial or financial relationships that could be construed as a potential conflict of interest.

## Publisher’s Note

All claims expressed in this article are solely those of the authors and do not necessarily represent those of their affiliated organizations, or those of the publisher, the editors and the reviewers. Any product that may be evaluated in this article, or claim that may be made by its manufacturer, is not guaranteed or endorsed by the publisher.
